# Managing and monitoring indoor air quality and surface decontamination in healthcare environments

**DOI:** 10.2478/aiht-2025-76-4013

**Published:** 2025-12-30

**Authors:** Giovanni Cappelli, Ilaria Rapi, Stefano Dugheri, Niccolò Fanfani, Veronica Traversini, Antonio Baldassarre, Anna Korelidou, Maali-Amel Mersel, Filippo Baravelli, Marinos Louka, Nicola Mucci, Lina Kourtella

**Affiliations:** University of Florence, Department of Experimental and Clinical Medicine, Florence, Italy; Link Campus University, Department of Life Science, Health, and Health Professions, Rome, Italy; Careggi University Hospital, Division of Occupational Medicine, Florence, Italy; EMBIO Diagnostics Ltd, Nicosia, Cyprus; Resysten Hungary Kft., Budapest, Hungary; CLASS srl, Budrio, Italy

**Keywords:** airborne contaminants, bacteria, European regulations, exposure risk mitigation, healthcare-associated infections, healthcare workers, patients, viruses, volatile organic compounds, bakterije, bolesnici, bolničke infekcije, europska regulativa, hlapljivi organski spojevi, lebdeće čestice, smanjenje rizika od izloženosti, virusi, zagađivala u zraku, zdravstveni radnici

## Abstract

Indoor air quality (IAQ) in healthcare facilities is a critical yet often underestimated factor associated with adverse health effects and increased risk of infectious outbreaks. Key pollutants include volatile organic compounds (VOCs), particulate matter, and various biological agents such as bacteria and viruses. While numerous variables contribute to IAQ, European regulations still have significant gaps, having historically focused more on individual substances than on the overall air environment. This review examines the most relevant IAQ parameters, current technologies available for their detection, and the regulatory landscape at the European level. Special attention is given to real-time monitoring systems. We also propose a concise operational guideline for IAQ management which combines continuous monitoring, evidence-based mitigation, and improvements to reduce exposure, increase resilience to airborne and surface threats, and produce measurable safety outcomes for patients and healthcare personnel within hospital settings.

## Abstract (hr)

Kakvoća zraka u zatvorenom u zdravstvenim ustanovama ključan je, a opet podcijenjeni čimbenik, povezan sa štetnim djelovanjem na zdravlje i povišenim rizikom od zaraza. Ključna su onečišćivala hlapljivi organski spojevi (engl. *volatile organic compounds*, krat. VOC), lebdeće čestice i razni biološki agensi, poput bakterija i virusa. Unatoč tomu što kakvoća zraka ovisi o mnogim varijablama, europska regulativa i dalje ima velikih nedostataka s obzirom na to da se kroz povijest primarno usredotočila na pojedine spojeve, a ne na njihov ukupni utjecaj na zrak. U ovom preglednom radu istražujemo najvažnije parametre kakvoće zraka u zatvorenom, postojeća tehnološka rješenja za njihovo otkrivanje i utvrđivanje te regulatorni krajobraz na europskoj razini. Posebna je pažnja posvećena sustavima nadzora i praćenja u stvarnom vremenu. Također predlažemo kratke radne smjernice za upravljanje kakvoćom zraka u zatvorenom koje objedinjuju stalno praćenje/nadzor i poboljšanja temeljena na dokazima koja bi trebala smanjiti izloženost, povećati otpornost na prijetnje iz zraka i s površina te predlažemo mjerljive ciljeve zaštite bolesnika i zdravstvenih radnika u bolničkom okružju.

Effective assessment and management of indoor air quality (IAQ) in healthcare facilities are critical to ensure patient and healthcare worker safety. Compromised air quality, whether through direct or indirect exposure, poses significant health risks to occupants and is associated with more than half a million deaths in Europe every year (1). IAQ is influenced by a range of interconnected factors, such as outdoor air quality, indoor activities, and occupant density. Given that individuals spend about 90 % of their time indoors (2), maintaining adequate IAQ is imperative to keep adverse health effects at bay.

Healthcare settings are no exception (3,4,5). Hospital environments are influenced by a variety of factors, including seasonal and weather conditions, the design and operation of ventilation systems, outdoor humidity and microbial loads, and the number of occupants and their activities. Low relative humidity and cold temperatures are believed to increase the transmission of respiratory viruses (6). Human activities like breathing, coughing, sneezing, talking, and laughing release viral aerosols into the indoor air (7). The transmission of SARS-CoV-2 and cluster cases are indeed primarily associated with confined, crowded, and poorly ventilated spaces (8), which, together with the vulnerable population and nature of healthcare activities, underscores the importance of managing IAQ effectively in a hospital environment.

Over the past decade, research has focused on understanding key aspects of IAQ in hospitals, such as identifying important pollutants and their concentrations, analysing their spatial distribution across different hospital areas (e.g., waiting rooms, wards, laboratories), determining seasonal variations, and exploring indoor-outdoor pollutant correlations due to external intrusions (9).

IAQ in healthcare facilities depends on the levels of a wide range of pollutants, including particulate matter (PM), volatile organic compounds (VOCs), formaldehyde (HCHO), carbon monoxide (CO), carbon dioxide (CO_2_), polycyclic aromatic hydrocarbons (PAHs), nitrous oxide (NO), and biological contaminants such as viruses, bacteria, pollen, microbial spores, and allergens. Poor IAQ in hospitals has been associated with outbreaks of infectious diseases and building-related illnesses, manifesting symptoms like headaches, fatigue, eye irritation, and other health issues among patients and staff (10). Their severity depends on the concentration of individual or combined pollutants and contaminants, their intrinsic toxicity, and the duration of exposure (9). To mitigate health risks, it is essential to implement effective policies and interventions in IAQ, supported by early identification of issues and comprehensive research.

However, the EU ambient air regulations (e.g., Directive 2008/50/EC and recent recasts on air quality) (11), which govern outdoor air limit values and establish policies for indoor exposures, have significant regulatory gaps in establishing standard levels for chemical, physical, and microbiological parameters of IAQ in healthcare facilities. Recent EU policy initiatives and Horizon projects have explicitly recognised the need for a harmonised indoor air agenda. France has introduced specific measures, including mandatory IAQ monitoring in healthcare settings, which commenced in 2024 (12). For the other EU countries, the absence of an integrated national policy remains a critical challenge, including the lack of specific legislative references, national guidelines, and standardised rules for data analysis and management of IAQ (13). In Italy, no single binding IAQ standard specific to healthcare buildings exists. Practice relies on sectoral guidance, regional regulations, professional standards, and international benchmarks, with academic reviews and national institutes calling for harmonised national rules and monitoring protocols (13). In the United Kingdom, healthcare ventilation and IAQ are addressed through technical standards and guidelines such as the National Health Service Health Technical Memorandum 03-01 A (14) and B (15). There where national standards are absent, an interim solution is to rely on the World Health Organization (WHO) guidelines or analogous standards such as those for ambient air quality. However, current heterogeneity of national standards and the reliance on a mix of guidelines, ambient air benchmarks, and technical memoranda underscore the need for a harmonised, evidence-based European guidance for IAQ in healthcare that would clearly specify pollutant thresholds, sampling and reporting protocols, and ventilation and filtration minima.

Biological pollutants have extensively been studied by numerous research groups, leading to guidelines and detailed protocols in several countries, such as those addressing *Legionella* (16, 17). The EU Good Manufacturing Practice specifies, for example, air cleanliness standards for medical device manufacturing, recommending a total airborne count limit of <1 CFU/m^3^ in Class A rooms, <10 CFU/m^3^ in Class B rooms, and <100 CFU/m^3^ in Class C rooms (18).

Microbiological monitoring and control of IAQ in hospitals is essential to preventing hospital-acquired infections (HAI) (19). Indoor air is predominantly contaminated by bacteria, moulds, and yeasts, and their concentrations are often used as indicators of a indoor environment health, as the presence of bio-aerosols can disrupt normal operation and compromise environmental safety (7, 20,21,22).

Factors such as temperature, relative humidity, and occupant density are closely linked to microbial spread. In this context, studies have shown that ventilation plays a critical role in regulating bio-aerosol levels within hospital environments, considering that bio-aerosols are estimated to account for 5–34 % of indoor air pollution ( 23,24,25).

The prevailing bacterial genera identified in hospital bio-aerosols are Gram-positive strains, such as *Staphylococcus* (e.g., *S. aureus*, *S. capitis*, *S. hominis*, *S. epidermidis*, and *S. warneri*) and *Micrococcus* (e.g. *M. luteus* and *M. lylae*), and Gram-negative strains, most notably those belonging to the genus *Neisseria* (26).

Fungi typically infiltrate buildings through heating, ventilation, and air conditioning (HVAC) systems, doors, or windows, but they also grow on building materials and furnishings. Fungal growth and sporulation are further facilitated by humidity (27). Fungal flora, particularly within HVAC systems, poses significant health risks by exacerbating allergies and symptoms of the sick building syndrome. It can cause mucous membrane irritation, fatigue, headache, vertigo, concentration and memory impairment, dermatosis, respiratory diseases, and even cancer (28). Moulds and fungi are also common triggers for asthma, allergic alveolitis, vasomotor rhinitis, and urticaria (hives) (29). The prevailing genera are *Penicillium*, *Aspergillus*, *Cladosporium*, and *Alternaria*.

Dancer (30) reports that methicillin-resistant *S. aureus* (MRSA) can survive on surfaces for as long as seven months, with an infectious dose as low as 4 CFU. *Clostridium difficile*, noted for its resilience, remains viable for over five months and has an infectious dose of mere five spores. The highly infectious norovirus can endure for up to seven days, with an infectious dose of fewer than 20 virions.

Investigations into the presence and concentrations of chemical pollutants in hospital environments have been relatively recent and limited to specific operational areas, which is why knowledge about chemical and physical pollution of indoor air in hospitals and its interaction with microbial pollution is still scarce. Chemical contamination in hospital settings often originates from cleaning, disinfectant, and sterilising agents containing substances such as ethylene oxide, glutaraldehyde, formaldehyde, alcohols, and anaesthetic gases or other agents used in medical procedures (31, 32).

Hospital indoor air is burdened by indoor VOCs, formaldehyde vapours, and particulate matter resulting from the frequent use of cleaning solutions and detergents, medical treatment, building furniture, and human activities, such as walking and movement, which resuspend PM in the air (29,30,31,32,33,34,35,36). Additional burden comes from outdoor air pollution with CO, PM, and ozone (O_3_) (35, 37). As regards outdoor ozone, it initiates the formation and emission of secondary organic aerosols and VOCs (e.g. terpenes and terpenoids), present as fragrances in a variety of cleaning products (38, 39).

Jung et al. (40) reported higher CO_2_ and VOC concentrations in inpatient rooms (4.61 g/m^3^ and 6.94 mg/m^3^, respectively) than in nurse stations, clinics, and waiting areas across 96 sites in 37 Taiwanese hospitals. One Chinese study of five inpatient buildings (41) revealed higher concentrations of phthalate esters in nurse stations, inpatient rooms, and doctors’ offices (20.66 µg/m^3^, 20.0 µg/m^3^, and 16.92 µg/m^3^, respectively) than in newly furnished houses, which points to a significant indoor air contamination.

These indoor air burdens call for further advancement of technologies for monitoring and managing IAQ in hospitals. While traditional systems focus on chemical, physical, and microbiological parameters, there is a growing need for sustainable control systems capable of measuring and, at the same time, suggesting adjustments to ventilation systems, cleaning and disinfection protocols, and operational procedures.

IAQ and building ventilation have long been assessed through CO_2_ concentrations, and levels exceeding 1.803 mg/m^3^ indicate compromised air quality (42). A technological advancement that can improve CO_2_ assessment in hospital units, especially those requiring highly focused staff, such as oncology units and operating theatres, are definitely the Internet of Things (IoT)-based sensor networks (43), as they integrate sensors, data collection devices, and network infrastructure. By leveraging grid computing and cloud technology, these networks enable real-time, efficient, and cost-effective IAQ monitoring. Furthermore, the integration of IAQ measurement systems with ventilation management systems allows for continuous feedback to optimise environmental conditions. Alarm and pre-alarm mechanisms can facilitate early intervention in cases of IAQ deterioration, enabling the implementation of specific mitigation procedures.

The aim of this narrative review is to synthesise current evidence on the main surface contamination and IAQ parameters relevant to hospital settings, evaluate the performance and applicability of smart, cost-effective monitoring technologies, and to critically appraise mitigation tools and practices used to control both airborne and surface contamination. It also focuses on real-time monitoring and data integration, current mitigation approaches, such as ventilation and HVAC management, disinfection technologies, automated sanitisation, and antimicrobial surface strategies to show the importance of integrating advanced monitoring systems with effective contamination control strategies to improve IAQ management in hospitals.

## LITERATURE SEARCH

With this aim in mind we ran a comprehensive search of electronic databases, including PubMed, Scopus, and Google Scholar using terms such as “indoor air quality”, “hospital”, “mitigation”, and “indoor air contamination”.

Our analysis included 200 full-text articles published in English between 2014 and 2025 as well as some older studies presenting interesting data not covered by more recent articles. We focused on articles on IAQ in the healthcare sector, and excluded those dealing with unrelated topics or those marginal to our interest, such as COVID-19 studies due to their narrow focus. Instead, we analysed research that took a broader perspective on hospital IAQ, aiming to provide evidence that is applicable to different healthcare settings.

Data from the selected studies were analysed qualitatively, and findings were divided into two main subjects, namely mitigation solutions and IAQ monitoring devices.

## MITIGATION SOLUTIONS TO AIRBORNE AND SURFACE CONTAMINATION

**Figure 1:**
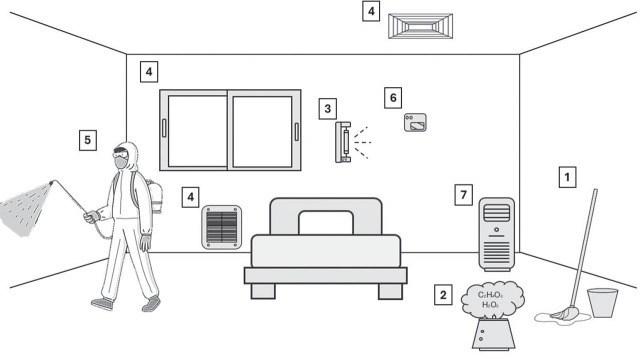
Indoor contamination mitigation strategies in a hospital setting:1) Cleaning activities and products; 2) Sanitisation with automated devices; 3) Ultraviolet germicidal irradiation (UVGI); 4) HVAC system; 5) Antimicrobial coatings; 6) Indoor air quality monitoring device; 7) Air purification unit

Figure 1 illustrates the complexity of indoor mitigation strategies that can be applied in a hospital setting, which encompass cleaning activities and products, automated sanitisation devices, ultra-violet germicidal irradiation (UVGI), HVAC system management, antimicrobial coatings, IAQ monitoring, and air purification. Ventilation systems play an important role in maintaining a safe and healthy environment. They require the use of air filters especially designed for hospitals, regular maintenance and monitoring of air filtration, and adequate rate of air exchange. The use of advanced technologies, such as UV light disinfection and anti-microbial coatings (AMC), has recently shown significant success in reducing microbial and chemical surface and air contamination (44). Cleaning practices have an important role in controlling the microorganism load (45) and chemical contamination, but the use of cleaning products should be closely monitored and adjusted to avoid even higher chemical burden on indoor air. Other effective interventions include rigorous hygiene protocols and educational campaigns to enhance healthcare workers’ awareness of appropriate hygiene practices.

### Cleaning activities and products

A growing body of evidence highlights the advantages of enhanced cleaning and decontamination in routine and outbreak scenarios (46). Cleaning protocols may be scheduled on an hourly, daily, or even twice-weekly basis. In addition, surfaces are cleaned when visibly soiled by spillages or on patient discharge (47). They are particularly rigorous in critical areas such as operating rooms, intensive care units (ICUs), transplant wards, and specialised “clean rooms,” where medications are prepared and handled under strict conditions of sterility.

Surfaces close to patients can be reservoirs of bacterial contamination, with concentrations ranging from a few to several hundred CFU/cm^2^. Yuen et al. (48) suggest that nosocomial pathogens such as MRSA, norovirus, *C. difficile*, vancomycin-resistant *Enterococcus*, and *Acinetobacter* species shed by patients can contaminate hospital surfaces at levels sufficient for transmission, and these pathogens also have the capacity to survive for extended periods and persist despite cleaning efforts. This in particular concerns objects that are frequently touched by patients and staff, such as telephones, door handles, faucets, light switches, levers, knobs, buttons, keyboards, push plates, and toys. Consequently, effective cleaning and disinfection practices for these surfaces are imperative in preventing cross-contamination and minimising the spread of HAI (46, 49). An effective method to manage bioburden is the use of detergent wipes on a daily basis, especially in acute-care wards (50). High-risk sites, such as ICUs, often require more frequent attention because of rapid recontamination potential of high-touch surfaces (51).

However, improper choice of detergents or application methods has the potential to compromise IAQ. The most straightforward method to mitigate the adverse effects of cleaning products is to reduce their usage, but such approach may not be a viable option in healthcare facilities upholding stringent hygiene standards. An alternative is to select cleaning products that emit minimal pollutants. For instance, floors are often treated with neutral or mildly alkaline detergents, such as chlorine derivatives, quaternary ammonium salts, or phenol-based solutions, while surfaces like furniture and windows are typically treated with neutral formulations containing alcohols or phenol compounds. Bathrooms are cleaned with mildly acidic detergents (52).

Debates continue around optimal cleaning methods, frequencies, equipment, and standards for surface cleanliness worldwide. Manual cleaning remains a prevalent method, though its execution and the substances used can differ significantly from one institution to another. The most common cleaning method is wiping, which relies on mechanical action to remove both organic debris and microorganisms (53). While this method is generally effective, it also carries the risk of transferring microorganisms to other surface areas if not executed carefully (54). Its efficacy depends on the properties of the wiping material (e.g., fabric structure and composition), pressure applied, motion geometry, number of passes, and specific microbial adhesion mechanisms (55). Wiping involves two types of action: mechanical, which removes microorganisms, and microbicidal action, (56) which either inhibits microbial growth or kills the pathogen.

There is a wide range of marketed biocidal products available to achieve that effect, including alcohols, chlorine compounds, aldehydes, peroxygens, and quaternary ammonium compounds (57). Each active ingredient has its own set of advantages and disadvantages. Alcohol, for instance, is both affordable and quick to kill bacteria, but its high volatility, flammability, corrosiveness to metals, and damaging effects on certain plastics limit its usability. Furthermore, it is not as effective against spores and certain viruses, which can be improved by adding salt (58). Chlorine-based disinfectants, such as hypochlorite, chlorine dioxide, and chloramine-T trihydrate, have become popular because of their effectiveness, affordability, and wide application range. Hypochlorite is favoured for its low cost, fast action, and broad efficacy, though it can be corrosive at high concentrations, easily inactivated by organic matter, and can irritate skin or release toxic gas in contact with ammonia or acids. Chlorine dioxide has a broad biocidal spectrum, including activity against mycobacteria, while chloramine-T trihydrate delivers prolonged bactericidal effects but may induce occupational asthma in chronic exposure (59). Hydrogen peroxide delivers strong germicidal activity, even against spores, and rapidly degrades, which makes it environmentally friendly. Improved versions are compatible with various materials, but the risk of chemical irritation remains. A similar mechanism of action is exhibited by peracetic acid, which acts swiftly against all microorganisms, even in the presence of organic matter and at low temperatures, without leaving residues. However, it is unstable when diluted and corrodes several metals (60). Quaternary ammonium compounds achieve good cleaning, deodorising, and antimicrobial effects, but their performance can be diminished by hard water and are less effective against Gram-negative bacteria and non-enveloped viruses (61).

Wiping techniques differ but all involve mechanical action combined with the application of disinfectant. The *spray and wipe* technique ensures adequate contact with the surface, but may result in overspray, missed areas, and the generation of airborne particles (55). The *dip and wipe* technique involves briefly immersing a wipe (5–10 s) in a disinfectant, wringing excess solution, and wiping a surface but an immersion too brief may not soak enough disinfectant and diminish its antimicrobial efficacy. The *soak and wipe* or bucket technique extends the soaking time ensuring a higher disinfectant load. However, prolonged soaking can enable chemical interactions that reduce antimicrobial activity and, if the wipe is reused, lead to cross-contamination (62). One promising solution to prevent wipe reuse and cross-contamination is to use ready-made, disposable disinfecting wipes, also referred to as pre-impregnated, pre-saturated, or pre-wetted disinfecting wipes, but prolonged storage may diminish antimicrobial activity due to degradation of active ingredients (63).

Finally, in order to achieve optimum outcomes with regard to decontamination and to minimise indoor air burden, some studies recommend opening windows for 10–15 minutes and that cleaning staff should receive a comprehensive training about the risks of exposure to chemicals, of accidents, and of contracting infections (64, 65).

### Sanitisation with automated devices

The idea of developing automated disinfection systems was to achieve superior decontamination with various microbicidal technologies, such as germicidal UV light, hydrogen peroxide, steam, and ozone. Whilst automated systems have been shown to enhance decontamination, they cannot be used as a substitute for routine cleaning procedures. Manual removal of organic matter, liquids, waste, and debris is imperative prior to the application of any disinfectant (66). Furthermore, the utilisation of such automated systems is typically confined to terminal or discharge cleaning, as some toxic disinfectants (such as hydrogen peroxide) and steam are not to be used around patients, and UV light is the most effective in unoccupied spaces (67). Terminal cleaning is generally done in emptied rooms, usually after a patient has been discharged. In the event of a known pathogen, the procedure is intensified to target it with appropriate disinfectant and its strength. The procedure commences at the uppermost level with dusting or wiping of lighting and other ceiling fixtures and ventilation components. The process then progresses downwards. Any items or equipment that are removed are meticulously cleaned with disinfectant before returning them to their original location. Furthermore, soft furnishings such as curtains, drapes, and screens are to be laundered whenever possible (30).

Steam vapour machines rapidly inactivate a broad range of pathogens, including vancomycin-resistant *Enterococcus* (VRE), MRSA, and Gram-negative bacilli like *Pseudomonas aeruginosa* (68). Overall, they cut hospital surface bioburden by more than 90 %. Although solid debris must be removed before disinfection, steam can be directly applied to soft and hard surfaces without prior cleaning. There is evidence that steam can be effective in both routine and outbreak cleaning. One study (69) showed that steam and a two-step water/detergent cleaning were similarly effective against MRSA, but steam excelled in removing adenosine triphosphate (ATP) from the surfaces and turned out to be a cost-effective, time-saving and environmentally friendly alternative to conventional cleaning methods.

However, concerns have been raised that routine steam cleaning in healthcare facilities is not best suited for decontaminating electric knobs, buttons, switches, and computer panels. Some hospitals now steam-clean commodes, non-electrical beds, and other furniture in nonclinical areas, as well as public toilets. However, because steam condensate causes slippages and damages surfaces, steam systems should only be used in well ventilated areas. Additionally, a comprehensive risk assessment is necessary to address the potential for aerosolised contaminants, although there is currently no evidence that this occurs (68).

Automated ozone decontamination effectively targets vegetative bacteria but is less effective against spores and fungi. Although ozone devices are inexpensive, its toxicity and corrosiveness for metals and rubber limits its application in healthcare facilities.

Hydrogen peroxide systems, delivered as vapours or dry aerosols, are effective for decontaminating hospital surfaces against a wide range of pathogens, including *Mycobacterium tuberculosis*, MRSA, viruses, spore formers, VRE, and multidrug-resistant Gram-negative bacteria. In high-risk wards, their use has been linked to significant reductions in infection rates with *C. difficile*. However, challenges remain: there is a risk of accidental human exposure, erosion of plastic and polymer materials, interference from residual organic debris, liquids, and waste (70, 71). Hydrogen peroxide disinfection systems are expensive, require skilled operators, and cannot be used in occupied rooms. Since a full cycle can take several hours, their use may interfere with cleaning schedules after patient discharge, as it can delay admission of new patients. Furthermore Pottage et al. (72) reported significant MRSA resistance to hydrogen peroxide on stainless steel, likely owed to its catalase activity breaking down hydrogen peroxide. As a result, such decontamination should be preceded by standard cleaning to remove bioburden.

### Ultra violet germicidal irradiation

Several considerations need to be addressed before integrating UV-C technology into routine hospital protocols, including cost, installation requirements, facility layout, integration with housekeeping services, operational management (e.g. lamp selection and longevity), and existing cleaning practices. In addition, UV light cannot penetrate surfaces. It does, however, penetrate cell walls and disrupts microbial reproduction (73, 74). UV-C light has a specific wavelength between 200 and 270 nm (usually 254 nm), which falls within the germicidal segment of the electromagnetic spectrum (200–320 nm). It is effective against airborne bacteria such as multi-drug resistant *M. tuberculosis* and the *Legionella* genus as well as against the measles virus (75) but is less effective against fungal spores.

UVGI has seen a growing application in hospitals (46), but its effectiveness decreases with the distance between the light source and contaminated surface and if the surface is soft and irregular. Boyce and Donskey (76) have found that hard smooth surfaces are ideal for UV-C decontamination.

Still, automated UV-C systems have several advantages, including effective sterilisation of bio-aerosols, ease of use, and shorter application (and therefore exposure) times (77). Recent studies report significant reductions in contamination with *C. difficile*, VRE, and MRSA on hospital surfaces, including keyboards and medical equipment outside patient rooms (78). However, surfaces should be pre-cleaned of organic matter to achieve optimum results (79, 80).

One study (81) has demonstrated that a portable pulsed UV light device for daily surface disinfection can halve housekeeping hours compared to manual disinfection using alcohol wipes.

The usual UV-C light dose of 8–13 mJ/cm^2^ can reduce bacterial load by 99.99 %, but, according to Dai et al. (82), a 65-second dose of 2.92 J/cm^2^ was required to achieve a 99.2 % reduction in *C. albicans*. Higher UV-C doses are also needed for fungi, due to their eukaryotic structure (83). In contrast, viruses are typically the most sensitive to UV light, although there are exceptions. In one study (84), it took a dose of 1048 mJ/cm^2^ (for 9 minutes at a distance of 3 cm) for complete inactivation of SARS-CoV-2. Speaking of doses associated with health risks, UV-C radiation can cause conjunctivitis at 5 mJ/cm^2^ and skin irritation at 10 mJ/cm^2^ and above.

Another UV disinfection method uses high-intensity narrow spectrum (HINS) light at 405 nm. Unlike UV-C, which breaks molecular bonds within the DNA, it photoexcites porphyrin molecules within bacteria, leading to the production of reactive oxygen species (ROS) that kill the pathogen (85, 86).

### HVAC system management

Agencies such as the American Society of Heating, Refrigerating and Air-Conditioning Engineers (ASHRAE), the World Health Organization (WHO), the Federation of European Heating and Air Conditioning Associations (REHVA), and the United States Centers for Disease Control and Prevention (CDC) recognise that effective ventilation plays a critical role in mitigating the transmission of airborne diseases. However, there are some controversies over the advantages and weaknesses of two major ventilation principles, namely mechanical (forced) and natural. HVAC systems, which rely on mechanical ventilation, circulate both fresh and recirculated air through ductwork, and research suggests that offices equipped with HVAC often exhibit lower levels of bacterial and fungal contaminants than those using natural ventilation (87, 88). In contrast, numerous studies have demonstrated that natural ventilation can effectively control the dissemination of airborne pathogens ( 89,90,91,92). In addition, Fonseca et al. (93) suggest that natural ventilation in healthcare facilities can better regulate relative humidity and CO_2_ levels.

Various institutions have established guidelines for ensuring adequate IAQ and occupant comfort in inpatient rooms. These recommendations include maintaining ambient temperatures between 21 and 24 °C, relative humidity levels of 40–60 %, and specific air velocities ranging from 0.05 to 0.20 m/s during heating and from 0.05 to 0.25 m/s during cooling (94,95). Furthermore, maintaining a stable differential pressure is recommended to prevent disturbances in the designed pressure gradient or airflow direction, which can be caused by routine activities such as door opening and elevator operation (96). Pressure gradient is achieved by creating a controlled imbalance between supply and exhaust airflows, ensuring that air moves predictably from cleaner to less clean spaces. This engineered gradient has been developed to reduce the risk of cross-contamination by unidirectional airflow across doorways and penetrations (97).

Hospital HVAC systems are specifically engineered for healthcare settings rather than generic confined spaces, such as offices or living areas to adjust ventilation to specific activities (98). This is of particular importance in high-risk areas such as operating theatres and delivery rooms, where 100 % outdoor air supply is essential. Furthermore, in environments where pollutant concentrations closely correlate with occupancy, outdoor air intake should be determined on a per-occupant basis to maintain acceptable IAQ.

To enhance the performance of HVAC systems, panel filters are routinely incorporated into ductwork in strict adherence to established standard guidelines. For optimal function and longevity high-efficiency particulate air (HEPA) filters are recommended, which require periodic cleaning and replacement (99). A robust filtration system typically employs two stages. Prefilters with a dust spot efficiency of 25–30 % are installed upstream of the cooling/heating coil to remove larger particulates and ensure clean heat transfer. The second stage involves a final filter with a minimum efficiency of 90 %, which is designed to capture nearly all fungal spores (2–5 µm in diameter) and bacterial colony-forming units (≥1 µm). It is important to note that these filters have limited success in capturing smaller biological agents, which have been observed to regrow under conditions of favourable humidity (100). In critical areas, such as those serving immunocompromised patients, the use of nanofiber filters is recommended to ensure a higher level of air purity. Examples of such filters include ultra-low particulate air (ULPA) filters, which efficiently remove 99.999 % of 0.1–0.2 µm particles (101). Some HVAC system integrate electrostatic filtration as highly effective yet requiring minimal maintenance, but its effectiveness may be diminished under high load and result in the generation of ozone and nitric oxide as by-products (102).

Some critical healthcare settings rely on multilayer filtration systems, typically comprising a prefilter, carbon filter, antibacterial filter, and HEPA filter. Recent research is increasingly focused on innovative promising techniques like biofilter adsorption (103).

In order to design an efficient ventilation system that effectively reduces contaminant concentrations in hospital rooms, it is necessary to address several key considerations. Firstly, the supply of outdoor air should be adjusted to specific occupancy levels and activities. Secondly, the filtration system must meet minimum efficiency criteria, i.e., the first-stage filter should provide moderate to high efficiency and the second-stage filter high efficiency, while maintaining an air exchange rate of approximately six air changes per hour as recommended by the ASHRAE guidelines (104). Thirdly, ventilation must be distributed evenly across breathing zones, and, where feasible, contaminated air should be actively extracted through dedicated extraction systems. Furthermore, HVAC systems should be strictly maintained through regular cleaning, monitoring, and filter replacement. The frequency of these maintenance procedures should be tailored to operating hours and occupancy levels (105).

### Antimicrobial coatings

At any time after disinfection, recontamination inevitably occurs, especially of frequently touched surfaces. Surface disinfectants can inactivate bacteria and viruses by several log_10_ steps over a few minutes, but these effects vanish as soon as the application is over (30). To problem can be overcome with antimicrobial coatings, which may contain an active biocidal agent anti-adhesives, or contact-or UV light-activated molecules to counteract surface contaminants (106).

Antimicrobial coating technologies have a long-lasting action and complement standard disinfection measures (30). However, their efficacy diminishes over time due to mechanical abrasion or elution.

Anti-adhesive surfaces reduce microbial attachment by minimising adhesion forces. For example, antibacterial fluorinated silica colloid super-hydrophobic surfaces decrease the adhesion of *S. aureus* and *P. aeruginosa* by about 64 % (107), but their overall antimicrobial efficacy is lower than with other coatings.

Contact-active coatings exert antimicrobial effects without the release of biocidal agents (108). For example, water-soluble antimicrobial polymers, which are covalently attached to surfaces, kill microbes *in situ* by disrupting bacterial cell membranes through cationic action, and quaternary ammonium polymers are often applied on medical implants and catheters under wet conditions. In case of a copper coating, its antimicrobial action involves the passive diffusion of CuO particles or Cu ions into bacterial cells, where they promote ROS formation, impair peptidoglycan maturation, and displace essential metals like iron (109). Copper antimicrobial effectiveness depends on its physical form (ions versus nanoparticles), oxidation state, concentration at the contact interface, and environmental conditions. However, effective “contact killing” requires that a copper-coated surface is cleaned from oxides, wax, and the like (110), because its efficacy relies on direct surface-microbe interaction, and any soiling under real-life conditions can significantly affect performance. Even with clean surfaces some authors (111) have reported modest performance and concluded that the overall benefits of copper coating remain uncertain (111).

More popular is coating with silver as a prominent biocidal agent, whose antimicrobial effects are owed to silver nanoparticles (Ag-NPs) and Ag-ions, though the primary mechanism of action remains unclear (112). One suggested mechanism is that Ag-NPs in direct contact with bacterial cells disrupts their membrane while another is that the coating releases Ag-ions that penetrate membranes, degrade chromosomal DNA, and disrupt cytoplasmic proteins (113). The efficacy of Ag-NPs and Ag-ions depends on their fluid coating medium.

ZnO nanoparticles also exhibit antimicrobial activity against Gram-positive bacteria through mechanisms such as ROS production and membrane disruption, achieving reductions of approximately 7 log_10_ for *E. coli* and *S. aureus* under wet conditions (114). Transparent ZnO coatings prepared with the polymer-salt method also exhibit bactericidal activity against both Gram-positive *S. aureus* and Gram-negative *E. coli* under natural light and dark conditions (115).

ROS can be generated not only via chemical pathways (e.g. Cu ion release) but also through photocatalysis. The most common photocatalyst in antimicrobial coatings is titanium dioxide (TiO_2_). Upon absorbing UV radiation in the 250–400 nm range, TiO_2_ reacts with water and oxygen to produce hydroxyl and superoxide radicals (116). Because these radicals have a very short lifespan, effective microbial inactivation requires close contact between the microorganisms and the photoactivated TiO_2_ surface (117). One study (118) reported that TiO_2_ nanoparticles achieved the highest bacterial inhibition against *P. aeruginosa.* However, typical indoor environments provide limited UV exposure since common artificial light sources emit little UV radiation. To overcome this limitation, TiO_2_ can be doped with small amounts of metals such as silver, copper, iron, or manganese to enhance its indoor photocatalytic activity. Another common method is to form heterojunctions with other photocatalysts, like carbon-based materials, which can improve charge separation. One such combination is g-C_3_N_4_/TiO_2_/Ag, which has been reported to significantly enhance antibacterial activity against the Gram-negative *E. coli,* having reduced its viability by 84 % after 2 h of visible light exposure (119). Another approach was reported by Krumdieck et al. (120), who utilised a scalable vapour deposition method to create a nanostructured solid 10 µm composite coating consisting of anatase and rutile phases of TiO_2_ combined with carbon. On stainless steel its photocatalytic activity achieved a reduction of more than 3 log in viable *E. coli* after 4 h of visible light exposure.

The use of visible light to generate ROS offers promising applications for indoor antimicrobial coatings, particularly in healthcare settings. This photodynamic mechanism employs a photosensitiser that absorbs visible light and transfers energy through its triplet state to molecular oxygen, resulting in ROS formation. Among these, singlet oxygen, located 0.98 eV above the oxygen ground state (121) plays a crucial role by offering three key advantages: it is generated by visible light, diffuses easily from the coated surface to target microorganisms (122), and its action is limited to a thin reactive layer of a few millimetres due to rapid deactivation through collisions with air molecules, which renders it environmentally safe beyond this range of action (123). In a six-month field study conducted in two hospitals, the application of a photodynamic antimicrobial coating on surfaces surrounding patients significantly reduced microbial burden compared to uncoated controls (122).

The durability of antimicrobial coatings was demonstrated when a combination of quaternary ammonium silyl oxide and titanyl-oxide moieties was applied via electrostatic spray to all surfaces in a hospital ICU in California (124). A single application reduced all bacterial levels by over 99 % (2 logs), and no antibiotic-resistant bacteria were detected for up to eight weeks, plus the bacterial counts did not return to pre-treatment levels throughout the 15-week monitoring.

This type of treatment is also promising for fabrics. One study (125) investigating TiO_2_-coated textiles for potential hospital use found that TiO_2_ coating retained most of its fabric bonding durability after 30 wash cycles at 40 °C, but its antimicrobial effectiveness dropped with higher washing temperatures. The coating was the most effective against MRSA. Washing did not significantly diminish its antibacterial activity; after 40 minutes of incubation, washed and unwashed samples performed similarly, and nearly 100 % of MRSA was eliminated after 60 minutes of UVA exposure.

Further research into antimicrobial coatings will hopefully inform important decisions as regards application, cost-effectiveness, durability, environmental conditions, safety, and potential bacterial resistance. Identifying the role of antimicrobial coatings in reducing hand contamination and cross-transmission will also help their integration in hospitals. With the development of standardised guidelines, antimicrobial coatings may become a valuable complement to the existing HAI-prevention strategies.

### Indoor air quality monitoring

The WHO updated its global air quality guidelines in 2021, establishing more stringent thresholds for key pollutants to better protect public health (126). These guidelines serve as a benchmark for governments and organisations worldwide to assess and manage air quality. The thresholds represent concentrations below which adverse health effects are unlikely to occur, even among sensitive populations.

Recently, there have been significant advancements in methods and strategies for monitoring air pollution, especially with the integration of real-time monitoring capabilities (127). The use of low-cost devices, either independently or alongside high-precision instruments, has expanded rapidly. Today’s market offers a diverse range of affordable IAQ monitoring systems equipped with cutting-edge sensor technologies, intuitive user interfaces, and robust platforms for device management (128). These modern solutions enable continuous, high-resolution temporal tracking of environmental parameters and airborne contaminants, while also supporting efficient data collection, processing, and visualisation to facilitate timely decision-making and responsive air quality interventions (129).

One of the most valuable features of modern IAQ monitoring devices is the integration of alert and notification systems within companion mobile applications and cloud-based dashboards. These systems are designed to provide real-time feedback, enhance user awareness, and support timely interventions in response to deteriorating air quality (130). For commercial and institutional settings, platforms like the NEMo (Ethera, Crolles, France) and airbeld™ (EMBIO Diagnostics, Nicosia, Cyprus) offer web-based dashboards with real-time data visualisation and alert management features (131). Alerts may appear as pop-up warnings, red status icons, or summary logs and can be configured to trigger emails or SMS messages to designated recipients. These dashboards also allow historical data analysis, which helps to correlate poor air quality events with specific activities or environmental conditions (132). In addition, some advanced devices are integrated with building management systems and IoT platforms, which automatically trigger HVAC adjustments, air purifiers (133), or other ventilation systems based on sensor readings.

Table 1 shows a selection of key IAQ monitoring devices along with their specific characteristics, including measured parameters and pollutants, physical dimensions, and data visualisation capabilities. Size, portability, data display, storage capabilities, and affordability are among the most important considerations when selecting a device for monitoring IAQ in both professional and residential settings. These devices have already been implemented in various IAQ studies, including those conducted in hospital settings. For instance, Palmisani et al. (134) conducted a study in two European hospitals utilising low-cost infrared LED particle monitors to measure PM, along with a photo-ionisation detector for monitoring VOCs, temperature, and relative humidity to identify critical factors influencing IAQ within oncology wards.

**Table 1:** List of the indoor air quality monitoring devices with their main features

**Ref.**	**Device**	**Measured parameters**	**Range**	**Portability**	**Configuration(dimension-mm)**	**Temporal data resolution**	**Remote management**	**Screen**
(165)	Aranet4 Home SAF Tehnika, Riga, Latvia	CO_2_, temp., RH %	CO_2_ 0–18.0 g/m^3^ Temp. 0–50 °C RH % 0–85 %	High (battery)	Station (70×70×24)	<1 min	Yes (Bluetooth, app)	Yes
(166)	View Plus Airthings, Oslo, Norway	Radon, PM_2.5_, CO_2_, temp., RH %, VOC	Rad 0–20000 Bq/m^3^ CO_2_ 0.72–9.0 g/m^3^ PM2.5 0–1000 μg/m^3^ Temp. 4–40 °C RH % 0–85 %	High (battery)	Station (170×90×33)	2.5–5 min	Yes (Wi-Fi, app)	Yes
(167)	Smart Indoor AQ Monitor Netatmo, Boulogne-Billancourt, France	CO_2_, RH %, temp., Noise	CO_2_ 0–9.0 g/m^3^ RH % 0–100 Temp. 0–50 °C Noise 35–120 db	Medium (power supply)	Station (45×45×155)	5 min	Yes (Wi-Fi, app)	No
(168)	Foobot Foobot, Paris, France	PM_2.5_, TVOC, temp., RH %	PM_2.5_ 0–1300 μg/m^3^ Temp. 15–45 °C RH % 30–85 % TVOC 0.54–2.60 mg/m^3^	Medium (power supply)	Station (172×71)	5 min	Yes (Wi-Fi, app)	No
(169)	uHoo uHoo, Singapore	NO_2_, O_3_, NO_2_, SO_2_, HCHO, NH_3_, H_2_S, O_2_, PM_1_, PM_2.5_, PM_4_, PM_10_, temp., RH %, CO_2_, CO, TVOC, air pressure	Temp. 40–85 °C PM 0–1000 μg/m^3^ TVOC 0–260 mg/m^3^ CO_2_ 0.72–18.0 g/m^3^ CO 0–1.15 g/m^3^ HCHO 0–2.46 mg/m^3^ NO_2_ 0–9.42 mg/m^3^ O_3_ 0–9.82 mg/m^3^ NH_3_ 0–13.9 mg/m^3^ SO_2_ 0–131 mg/m^3^ H_2_S 0–139 mg/m^3^ O_2_ 0–30 %	Medium (power supply)	Station (200×180×57)	1 min	Yes (Wi-Fi, app)	No
(170)	TSI IAQ TSI, Shoreview, MN, USA	CO_2_, CO, NO_2_, SO_2_, O_3_, HCHO, PM, temp., RH %, VOC	CO 0–1.15 g/m^3^ NO_2_ 0–37.7 mg/m^3^ SO_2_ 0–52.4 mg/m^3^ O_3_ 0–19.6 mg/m^3^ VOC 0–1885 μg/m^3^ Temp. 0–60 °C RH % 0–100 % CO_2_ 0–3.61 g/m^3^ HCHO 0–1.23 mg/m^3^ PM 0–1000 μg/m^3^	Medium (power supply)	Station (84×178×44)	1 sec	Yes (PC or app)	Yes
(171)	Ethera NEMo Ethera, Crolles, France	HCHO, CO_2_, LVOC, temp., RH %, air, pressure, NO_2_, O_3_, PM_1/2.5/10_	HCHO 0–0.025 mg/m^3^ CO_2_ 0–9.0 g/m^3^ LVOC 0.13–21.7 mg/m^3^ Temp. 55–125 °C RH % 0–99 % PM 0–1000 μg/m^3^	High (battery)	Station (180×120×65)	10 min	Yes (app)	No
(172)	airbeld™ Embio Diagnostics, Nicosia, Cyprus	Temp., RH %, CO_2_, PM_1/2.5/4/10_, VOC, NO_×_	Temp. 10–50 °C RH % 0–90 % NO_×_ 0–942 mg/m^3^ VOC 0–2.17 g/m^3^ CO_2_ 0–0.90 g/m^3^ PM 0–1000 μg/m^3^	Medium (power supply)	Station (117×112×39)	1 sec	Yes (Wi-Fi, app)	No
(134)	Corvus IAQ Monitor ION Science Ltd, Royston, UK	VOC, temp., RH %	VOC 0–0.217 g/m^3^ Temp. −40–125 °C RH % 0–99 %	Medium (power supply)	Station (68×176×123)	1 min	Yes	No
(173)	PCE-AQD 20 Capannori, Italy	CO_2_, temp., RH %	CO_2_ 0–72.1 g/m^3^ Temp. 0–50 °C RH % 0–80 %	High (battery and power supply)	Station (130×90×40)	<5 sec	No app, datalogging	Yes
(174)	Airthings for Business-Space Pro Airthing, Oslo, Norway	CO_2_, Temp., RH %, VOC, PM_2.5_, radon, virus	CO_2_ 0.72–9.0 g/m^3^ PM2.5 1–1000 μg/m^3^ VOC 0–43.4 mg/m^3^ Temp. 4–40 °C RH % 0–85 %	High (battery and power supply)	Station (170×90×33)	<5 sec	Yes (app)	Yes
(175)	AirVisual Pro IQAir, Goldach, Switzerland	PM, temp., RH %, CO_2_	PM 0–1000 μg/m^3^ Temp. −40–90 °C RH % 0–100 % CO_2_ 0.72–18.0 g/m^3^	High (battery)	Station (82×184×100)	<5 sec	Yes (app)	Yes
(176)	Atmotube Pro Atmotube, San Francisco, CA, USA	TVOC, temp., RH % PM	TVOC 0–260 mg/m^3^ PM 0–1000 μg/m^3^ Temp. 0–65 °C RH % 0–100 %	High (battery)	Station/Wearable (86×50×22)	1 min	Yes (app)	No
(166)	Airbird Airbird, Copenhagen, Denmark	CO_2_, temp., RH %	CO_2_ 0–9.0 g/m^3^ Temp. 10–60 °C RH % 0–100 %	High (battery)	Station (229.5×99.5×95)	10 min	Yes (app)	No
(177)	HALO 3C Smart Sensor HALO Smart Sensors IPVideo, Bay Shore, NY, USA	CO_2_, TVOC, PM, temp., RH %	CO_2_ 0.72–18.0 g/m^3^ TVOC 0–43.4 mg/m^3^ PM2.5 0–1.000 μg/m^3^ Temp. 10–60 °C RH % 0–100 %	Medium (power supply)	Station (127×43)	<5 sec	Yes (app)	No
(166)	Awair Element – Omni Awair, San Francisco, CA, USA	CO_2_, TVOC, PM_2.5_, temp., RH %	PM2.5 0–1.000 μg/m^3^ CO_2_ 0.72–9.0 g/m^3^ TVOC 0–26.0 mg/m^3^ Temp. 10–50 °C RH % 0–100 %	Medium (power supply)	Station (160×90×40 – 98×98×34)	Seconds	Yes (app)	No
(166)	Eve Room Eve Systems GmbH, Munich, Germany	TVOC, temp., RH %	TVOC 0–260 mg/m^3^ Temp. 0–50 °C RH % 0–100 %	High (battery)	Station (54×54×15)	Seconds	Yes (app)	Yes
(178)	Blueair Aware Blueair, Stockholm, Sweden	PM_2.5_, VOC, CO_2_	PM2.5 0–1.000 μg/m^3^ TVOC 0–43.4 mg/m^3^ CO_2_ 0.72–18.0 g/m^3^	Medium (power supply)	Station (170×100×100)	Seconds	Yes (app)	No
(179)	QP Pro 2 Air Quality Monitor SmartAir, Bejing	PM, VOC, CO_2_, Temp., RH %, noise	Temp. 10–60 °C RH % 0–100 % CO_2_ 0.72–18.0 g/m^3^ PM2.5/10 0–999 μg/m^3^ TVOC 0–2.17 g/m^3^ Noise 36–95 db	High (battery)	Station (74×85×104)	Seconds	Yes (Wi-Fi)	Yes
(166)	Sensedge Go Mini IAQ Monitor Kaiterra, San Francisco, USA	PM_2.5_, PM10, TVOC, temp., RH %, CO_2_, NO_2_	PM 0–1000 μg/m^3^ TVOC 0–4.34 g/m^3^ CO_2_ 0.72–3.61 g/m^3^ NO_2_ 0–18.8 mg/m^3^ Temp. −20–100 °C RH % 0–100 %	High (battery and power supply)	Station (155×126×35)	1 min	Yes (app)	No
(180)	Temtop M2000 Elitech Technology, Inc, Silicone Valley, USA	PM_2.5_, PM_10_, HCHO, CO_2_	PM 0–999 μg/m^3^ CO_2_ 0–9.0 g/m^3^ HCHO 0.00123–6.15 mg/m^3^	High (battery)	Palm-top (73.5×223.5×37.5)	<1 min	No	Yes
(181)	Aeroqual Series 200/300/500 Aeroqual, Auckland, New Zealand	O_3_, NO_2_, SO_2_, CO, CO_2_, PM_2.5_, PM_10_, temp., RH %	Temp. 40–124 °C RH % 0–100 % CO_2_ 0–9.0 g/m^3^ CO 0–1.15 g/m^3^ SO_2_ 0–262 mg/m^3^ NO_2_ 0–1.88 mg/m^3^ O_3_ 0–58.9 mg/m^3^ PM 0.001–1000 μg/m^3^	High (battery)	Palm-top (data not available)	<1 min	Yes (app)	Yes
(182)	GrayWolf IAQ Meters GrayWolf Sensing Solutions, Shelton, CT, USA	PM_2.5_, PM_10_, CO_2_, CO, TVOC, HCHO, O_3_, radon	VOC 0–43.4 mg/m^3^ PM 0–1000 μg/m^3^ CO_2_ 0.72–9.0 g/m^3^ O_3_ 0–1.96 mg/m^3^ CO 0–5.73 g/m^3^ HCHO 0–1.23 mg/m^3^	High (battery or power supply)	Palm-top (data not available)	<1 min	Yes (app)	Yes
(183)	Testo 400 Testo SE & Co. KGaA, Lenzkirch, Germany	CO_2_, CO, temp., RH %	CO_2_ 0–18.0 g/m^3^ CO 0–0.115 g/m^3^ Temp. 20–70 °C RH % 0–100 %	Medium (power supply)	Palm-top (210×95×39)	Seconds	Yes (app)	Yes
(143)	Honeywell IAQPoint2 Honeywell Analytics Inc., Lincolnshire, IL, USA	CO_2_, TVOC, temp., RH %	CO_2_ 0–3.61 g/m^3^ TVOC 0–4.34 mg/m^3^ Temp. 10–60 °C RH % 0–100 %	Medium (power supply)	Palm-top (163×77×38)	Seconds	Yes (app)	Yes
(184)	SI-AQ 110 Sauermann Sauermann Industries SAS, Chevry-Cossigny, France	CO_2_, CO, NO_2_, NO, SO_2_, H_2_S, HCHO, temp., RH %	CO_2_ 0–9.0 g/m^3^ CO 0–0.229 g/m^3^ NO_2_ 0–37.7 mg/m^3^ NO 0–471 mg/m^3^ SO_2_ 0–52.4 mg/m^3^ H_2_S 0–139 mg/m^3^ HCHO 0–12.3 mg/m^3^ Temp. −40–125 °C RH % 5–95 %	High (battery and power supply)	Palm-top (279×254×102)	Seconds	No app, Yes data logging	Yes
(143)	ARW-HD21AB ARW Misure, Zanè, Italy	CO_2_, CO, temp., RH %, air pressure	CO_2_ 0–9.0 g/m^3^ CO 0–0.229 g/m^3^ Air pressure 800–1100 hPa	Medium (power supply)	Palm-top (210×90×40)	Seconds	No app, Yes data logging	Yes

App – application software; PM – particulate matter; RH % – relative humidity; Temp. – temperature; TVOC – total volatile organic compounds; VOCs – volatile organic compounds

Portability in real-time continuous monitoring is also an important factor. IAQ monitors listed in Table 1 are mainly plug-in devices, even though they are small and lightweight. Considering that free electric sockets are often in short supply, this may be a problem, especially as monitoring systems should be placed as far away from heat and cooling sources as possible, including windows, HVAC outlets, fans, and doors (135). Furthermore, plugged-in device can be disconnected accidentally and lose data. This is why battery-powered devices, generally lithium-ion based, may have an advantage and be more suitable for monitoring work environments, provided, of course, that the batteries are recharged and replaced regularly (136).

Most small devices, such as the Temtop M2000 (San Jose, CA, USA), are hand-held and mainly detect leaks, but can also be used in a fixed position with a special base. Others, such as the HALO Smart Sensor (IPVideo, Bay Shore, NY, USA) or Blueair Aware (Blueair, Stockholm, Sweden) are small stations with a fixed base that can be mounted on the wall.

Other factors to consider is the data interface and the storing capacity. Some devices like as airbeld™ or NemoEthera send data to a computer dashboard, which can display historical data and trends, informing necessary changes to improve IAQ (137). Today, most devices are paired with a mobile application to visualise data, trends, and history, and some tools, such as airbeld™, Airthings View Plus (Airthings, Oslo, Norway), or Awair Omni (Awair, San Francisco, CA, US) offer a system of alerts in the event of IAQ deterioration and suggestions to restore optimal conditions (138).

An interesting aspect to consider is the variation in how different devices provide feedback. Some devices use a blinking or red warning light to signal poor IAQ, while others rely on numerical displays (139). In some cases, users may not be experts on the subject and may not understand what the numbers displayed mean, especially if there is no clear explanation. For example, a PM_2.5_ level of 35 µg/m^3^ may seem alarming, if no context is provided to make it clear that this is within acceptable air quality limits (140).

One important thing to consider is whether the IAQ monitoring device has been certified by an internationally recognised institution or standard (141), such as the International WELL Building Institute or the RESET Air (GIGA, Shanghai, China). These certifications ensure compliance with performance-based air quality benchmarks (142, 143).

One of the key benefits of these devices is their significantly lower cost compared to standard or reference instruments, allowing users to purchase multiple units to monitor specific environments and cover more indoor areas. Such detailed monitoring enables users to gain a more comprehensive understanding of IAQ in the areas where they spend a significant amount of time (144).

Sensor measurement accuracy is imperative for making informed decisions based on its data (145). However, sensor performance can vary dramatically over time and space, as the surrounding environmental conditions change (146). Precision can also vary between same-model devices. The accuracy of measurements by a single device can depend on a variety of factors, from calibration to its location or seasonality (147,148,149,150,151). Limitations such as lower accuracy, data reproducibility, significant inter-sensor variability as well as susceptibility to environmental factors, humidity in particular, still seem to bother low-cost sensor technology and may outweigh their other benefits (152). One study (153) has shown that high relative humidity can result in overestimated particulate matter concentrations, while low humidity may lead to underestimation. However, these imprecisions can be addressed by established calibration protocols and algorithms, suggest the authors.

We would also like to address the issue of limited capability for microbiological air monitoring with these devices, as only the manufacturer of Airthings for Business-Space Pro claims the ability of its device to measure the presence of airborne viruses but provides no information about the mode of detection. As current microbiological standards only cover surface contamination (154), one alternative for direct air monitoring of biological contamination could involve indirect monitoring of surfaces with luminometers, particularly those based on ATP bioluminescence. ATP luminometers use different reference parameters, depending on the model and the environment to be monitored. Reference levels range from 100 to 500 relative light units (RLU) per 100 cm^2^ of a sanitary area (155). Some studies suggest, however, that some luminometers may not be sensitive enough to detect very low microbial contamination (156, 157). Furthermore, ATP readings can be greatly inflated by disinfectants, microfibre products, food, and drink spills, and synthetic plastics used in cleaning and laundry services (158). Because of these drawbacks, ATP monitoring is perhaps more useful for screening (159).

Several recent epidemiological studies have established an association between air temperature, relative humidity, and the transmission of microorganisms. Sundell et al. (160) report a positive association between low outdoor temperature plus low indoor relative humidity and high weekly incidences of influenza, while Kim et al. (161) report a significant association with the concentrations of airborne bacteria and moulds, which varied with seasonal changes and buildings. Another study (162) indicated that dry indoor conditions typical of winter lead to slower inactivation of viruses, increasing the concentration of active virions in poorly ventilated spaces. These findings provide actionable insights for regulating indoor bio-aerosol levels through targeted adjustments of temperature and humidity across diverse building types.

Besides temperature and humidity, one review article (163) also suggests monitoring carbon dioxide (CO_2_), temperature, humidity, and particulate matter to assesses the risk of airborne virus transmission in indoor environments like schools, offices, and commercial establishments. Another study (162) indicated that dry indoor conditions typical of winter led to slower inactivation of enveloped viruses like SARS-CoV-2, increasing the concentration of active virions in poorly ventilated spaces. These studies collectively underscore the critical role of temperature and humidity in influencing indoor bio-aerosol levels and demonstrate the effectiveness of environmental monitoring tools in assessing and managing the risk of airborne viral transmission in various indoor settings.

In conclusion, low-cost sensors remain strategically valuable for tracking sources of IAQ pollution. Their affordability and portability make them suitable for large-scale monitoring projects, enabling the collection of high-density spatial and temporal data. Advancements in calibration techniques, such as the use of machine learning algorithms, have shown promise in enhancing the accuracy and reliability of low-cost sensors. Furthermore, integrating light and noise measurements (already existing in some IAQ monitoring devices mentioned above) could complement the understanding of healthy IAQ, since noise and improper lighting are associated with visual fatigue, stress, headaches, sleep disorders, and irritability in hospital staff and patients (184).

## CONCLUSION

This review underscores the importance of cutting-edge contamination mitigation procedures and of IAQ monitoring to ensure the well-being of healthcare personnel and patients. It is, however, limited in scope. While the focus is on healthcare facilities, we deliberately excluded studies on SARS-CoV-2 contamination control to consider a wider range of contamination scenarios. Moreover, due to an absence of sources, there is an imbalance between the more abundant and detailed studies on biological surface contamination and the scarcer ones on chemical pollutants and their effects on IAQ in hospitals. Furthermore, we have reviewed only the most readily available monitoring and mitigation solutions and mostly disregarded experimental technologies, which are less accessible to hospital settings.

Regardless of these limitations, our review proposes a novel framework for IAQ management that encompasses continuous surveillance with evidence-based mitigation and quality improvement. The objective of this framework is to provide facility managers and clinicians with a roadmap for reducing various types of exposures, strengthening resilience against airborne and surface threats, and generating measurable outcomes that will guide the gradual improvement of healthcare environment safety.
